# The Assessment of Problematic Internet Pornography Use: A Comparison of Three Scales with Mixed Methods

**DOI:** 10.3390/ijerph17020488

**Published:** 2020-01-12

**Authors:** Lijun Chen, Xiaoliu Jiang

**Affiliations:** Department of Psychology, School of Humanities and Social Sciences, Fuzhou University, Fuzhou 350108, China; psyjxl@126.com

**Keywords:** problematic pornography use, internet pornography use, problematic pornography consumption scale, problematic pornography use scale, the short internet addiction test adapted to online sexual activities

## Abstract

The primary aim of this study was to compare different screening tools for problematic internet pornography use (IPU) and identify the most accurate measure. The reliability and validity of three scales, namely, the Problematic Pornography Consumption Scale (PPCS), Problematic Pornography Use Scale (PPUS), and Short Internet Addiction Test Adapted to Online Sexual Activities(s-IAT-sex), were examined using three homogeneous groups, respectively. A total of 972 adults (mean age = 24.8) from 28 provinces/regions in China participated in the quantitative part (QUAN). The Brief Pornography Screener served as the reference standard. The PPCS demonstrated stronger reliability and validity, including criterion validity, as well as greater sensitivity and acceptable specificity; therefore, it was considered to be the more accurate screening instrument. In the qualitative part (QUAL), we interviewed 22 volunteers and 11 therapists (who had worked with individuals with problematic IPU) to examine their perspectives on the core features of problematic IPU and dimensions of the PPCS. Almost all the interviewees endorsed the structure of the PPCS. These findings encourage the use of the PPCS in future research studies and underscore its screening applications because of its ability to classify IPU as problematic or nonproblematic.

## 1. Introduction

Internet pornography use (IPU) is a sexual behavior [1], corresponding to the use of internet to engage in various gratifying sexual activities also known as online pornography use or cybersex [2,3,4]. It comprises a variety of online sexual activities (OSAs), including watching pornography, online pornography exchange, engaging in sex chats, using sex webcams, searching for sexual partners, or engaging in sexual role playing, among which stands the watching pornography, which is the most popular activity [5]. According to the past findings, engaging in IPU sometimes derives various negative consequences, such as financial, legal, occupational, and relationship trouble or personal problems [6]. Feelings of loss of control and persistent use despite these adverse outcomes constitute compulsive cybersex or problematic IPU. To date, no consensus exists regarding the conceptualization and diagnosis of problematic IPU. For instance, numerous terms have been used to describe the phenomenon (e.g., internet sex addiction [7,8], problematic online sexual activities [9], cybersex addiction [10], and problematic internet pornography use [6]). Although these concepts are slightly different, they all comprise three crucial components: the medium (the internet), the content (sexual behavior), and the problematic use (the compulsive behavior). Regardless of the debate, it is now acknowledged that excessive involvement in IPU or cybersex may become dysfunctional and associated with addiction symptoms (e.g., loss of control, compulsive use). Considering these inconsistent terms sharing crucial components, problematic IPU may be regarded as a subtypes of problematic internet use from a classification perspective, which may help advance clinical and research efforts into its prevalence and impact.

Nevertheless, evidence regarding the problematic IPU is inconsistent, due to the heterogeneity of assessment tool. The fundamental reason is that the definition and diagnostic criteria of problematic IPU is still unclear. In order to address these conceptual ambiguities, researchers have developed several scales that measure different aspects of pornography use [11]. Some briefer scales are more convenient to administer, but they underscore the self-perceived addiction (e.g., Cyber-Pornography Use Inventory-9). Some of these scales have been designed to assess the motivations underlying pornography use among hypersexual men (e.g., Pornography Consumption Inventory) [12]. Some scales fail to capture the different aspects of problematic IPU and focus solely on specific dimensions (e.g., the Pornography Craving Questionnaire, PCQ). Additionally, some globally accessible websites host the Cybersex Addiction Test, Sexaholics Anonymity Test, Sex Addicts Anonymous, and Sexual Addiction Screening Test, which assess difficulties in exercising self-control, its negative consequences, and the social problems that are associated with sexual activities. Furthermore, assessing IPU, using measures of sexual addiction, entails a few challenges. Specifically, these assessments may not be able to capture the characteristics of the activities (e.g., chat-based cybersex, sexual video games that cannot be played offline) and symptoms (e.g., separation from reality due to immersion in the virtual world that are unique to IPU. To address this gap in the literature and conduct further research in this domain, assessments with strong psychometric properties are much needed [5,7].

Several scales of problematic IPU are available to researchers and clinicians. Indeed, a recent meta-analysis identified 22 psychometric instruments that assess problematic pornography use [11]. Otherwise, most of the studies that have been conducted during the past decade had used self-developed items and a few of these measures have been subsequently revalidated [4,5,13]. Therefore, it is difficult to compare the results of different studies because there is a lack of concordance in the assessments that have been used. In order to select suitable tools for comparison from the existing scales, a systematic review was conducted. The following terms and their derivatives were used in multiple combinations: (Cybersex* OR internet porn* OR hypersex*) AND (addict* OR compulsiv* OR problem*) AND (assessment OR scale OR instrument OR measure*), to identify relevant studies in order to address the questions related to assessment and available screening questionnaires. The selection criteria of the literature search were limited to articles focusing specifically on cybersex and/or internet pornography consumption and dysfunctional cybersex, and also describe the development and adaptation of self-reported psychometric instruments that assesses at least one aspect of problematic pornography use. Finally, we found a total of 27 instruments on assess the problematic IPU (cybersex). Through the systematic review process conducted, we decided to retain three scales that were developed to measure problematic pornography use, even if not all of the three scales were specifically designed to measure internet pornography, as a large majority of participants used online pornography, and the developers of these scales suggested that they could be used to measure problematic IPU [14,15], additionally we replaced “pornography” into “internet pornography” in the Chinese version. We selected these three scales for the following reasons: (1) they include fewer items and are thus easily administered measures, (2) all of them cover the core characteristics of IPU, such as loss control, (3) they are grounded in addiction components such as impaired control, conflict, salience [11], (4) they are applicable within the Chinese culture [16,17,18,19], and (5) they display strong test-retest (i.e., two weeks) reliability; consequently, these three previously validated scales were identified for further examination. First, the Short Internet Addiction Test Adapted to OSAs (s-IAT-sex), which has demonstrated satisfactory psychometric properties [9]. However, this scale has been validated only among men [5], and a large number of studies have shown that there are substantial gender differences in IPU [18,20,21]. Second, the Problematic Pornography Use Scale (PPUS) [15], which has been validated using a large sample; unfortunately, however, a valid cutoff score has not been specified for this measure. Third, the Problematic Pornography Consumption Scale (PPCS); this scale is founded upon the theoretical framework of Griffiths’s components model of addiction [22]. All three scales include strong internal consistency and a valid factorial structure, which has been supported by the results of confirmatory factor analysis (CFA) [9,14,15,19]. Nevertheless, it is difficult to compare the findings of studies that have used these scales because they entail different factor structures. Therefore, it is necessary to select reliable indicators and methods, and identify the most accurate instrument.

In order to effectively compare different scales, a unifying and reliable standard should first be established. The Brief Pornography Screener (BPS), which is a screening tool that measures loss of self-control, overuse of problematic pornography use, may be useful in identifying individuals who are at risk for problematic pornography use or can serve as a proxy measure [23]. Kraus et al., who developed the BPS, have proposed that the diagnostic criteria for compulsive sexual behavior (CSB) should be included in the new International Classification of Diseases (ICD-11) [24], and this proposal has been accepted. According to the upcoming ICD-11’s diagnostic criteria for impulse control disorder [25], patterns of failure to control intense sexual impulses or urges and the resultant repetitive sexual behaviors are considered to be the characteristic features of the disorder. The BPS considers compulsive pornography to be the core component of problematic pornography use. Moreover, the BPS has been used with different samples, and it has demonstrated satisfactory psychometric properties among American and Polish pornography users [26]. Many past studies have used the BPS to identify pornography addicts. Furthermore, it has also been used to ascertain the severity of problematic pornography use among men who seek pharmacologic or psychological treatment as a result of their loss of control over their sexual behaviors [27,28,29]. Therefore, in this study, the BPS scores were used as the reference standard against which the sensitivity and specificity of the three aforementioned scales were ascertained.

Several recent reviews have focused specifically on the conceptualization and assessment of problematic pornography use [4,11,30,31]. Some reviews have briefly summarized and commented on the included instruments [5], whereas others have evaluated their ability to assess the core components of problematic pornography use [11]. However, no past study has compared the different scales and identified the most accurate measure of problematic pornography use using a same standard or indicator. Measures of problematic IPU are heterogeneous, and each scale focuses on a different aspect of problematic IPU. Furthermore, because these scales have not been extensively validated, it is difficult to compare the findings of the studies that have used them. In addition, the sensitivity of the different scales that assess problematic IPU have not been adequately compared. Therefore, in the present study, a QUAN→QUAL mixed-methods design was conducted, including (1) using quantitative methods to identify a scale with a higher sensitivity index from three selected scales (PPCS, PPUS, s-IAT-sex) for assessing problematic IPU. Moreover, the duration of usage, frequency of engagement in OSAs, sexual compulsivity, and pornography cravings were used to examine the criterion validity of the assessments. Subsequently, (2) qualitative interviews were conducted with volunteers and therapists who have serviced the individuals in trouble of problematic IPU to further examine the appropriateness of the “more accurate” scale from the service providers’ perspectives, whereby the qualitative part helps to evaluate and interpret the results obtained from the main quantitative study.

## 2. The Quantitative Part: A Comparison of the Three Retained Scales

### 2.1. Materials and Methods

#### 2.1.1. Sample

The study sample consisted of 560 men and 412 women, and the mean age of the sample was 24.8 years [*standard deviation (SD)* = 7.2 years; range = 18–48 years]. The group comparisons of the demographic characteristics of the three study samples can be inferred from Table 1.

#### 2.1.2. Instruments

##### Three Main IPU Measurements

PPUS. The PPUS is a 12-item self-report scale that assesses four dimensions of IPU [15]: distress and functional problems, excessive use, difficulties in self-control, and IPU to escape or avoid negative emotions. In the Chinese version of the assessment, the term “pornography,” which was used in the original scale, was modified as “internet pornography” in all instances (e.g., “I spend too much time being involved in thoughts about internet pornography”). The participants were required to indicate the frequency with which they had engaged in IPU during the past 6 months on a six-point scale that ranged from 0 (never) to 5 (all the time). Higher scores were indicative of a greater severity of engagement in IPU. The Cronbach’s alpha of the total scale was 0.95 in this study.

PPCS. The PPCS was used to measure problematic IPU [14]. Responses were recorded on the following 7-point scale: 1 = never, 2 = rarely, 3 = occasionally, 4 = sometimes, 5 = often, 6 = very often, 7 = all the time. PPCS consists of 18 items, and assesses the six core components of addiction: salience, mood modification, conflict, tolerance, relapse, and withdrawal. Each factor is measured by three items (e.g., “I felt that I had to watch more and more internet porn for satisfaction” is an item of measure “tolerance”); the Cronbach’s alphas of the aforementioned six factors were 0.77, 0.84, 0.71, 0.78, 0.86, and 0.86, respectively, in the study. The Cronbach’s alpha of the total PPCS was 0.96. A cutoff score of 76 was used to ascertain normal and problematic use; specifically, scores that were greater than 76 were indicative of problematic use.

s-IAT-sex. Responses to each of the 12 items of the s-IAT-sex are recorded on a five-point scale that ranges from 1 (never) to 5 (always) [9]. The scale consists of two dimensions. The first factor assesses poor self-control and difficulties in reducing the amount of time that is spent online (six items, e.g., “How often do you find that you stay on Internet sex sites longer than you intended?”), whereas the second factor measures the functional impairments that are associated with engagement in cybersex (six items, e.g., “How often do you feel depressed, moody, or nervous when you are offline, which goes away once you are back on internet sex sites?”). The composite score, which can be computed by summing the individual item scores, can range from 12 to 60; higher scores are indicative of greater problems. The internal consistency (Cronbach’s alpha) coefficients of the total scale and first and second factors were 0.89, 0.77, and 0.88, respectively, in this study.

##### Criterion Validity Questionnaires

*PCQ*. This 12-item questionnaire is a unidimensional assessment [32,33]. The following are a few sample items: “If the situation permitted, I would watch pornography right now” and “If I were to watch pornography right now, I would have difficulty stopping.” The respondents were required to indicate how strongly they agreed with each item using the following seven response options (presented without numerals): “completely disagree,” “somewhat disagree,” “disagree a little,” “neither agree nor disagree,” “agree a little,” “somewhat agree,” and “completely agree.” Higher scores are indicative of a greater craving for pornography. The Cronbach’s alpha of this scale was 0.92 in the current study. The instructions of the PCQ present a craving-for-pornography vignette, which requires the respondent to imagine that they are alone in their room and seated in front of their computer and that they have a strong urge to watch their favorite type of pornography.

The Sexual Compulsivity Scale (SCS). The extent to which participants exhibit the characteristics of compulsive pornography use was assessed using the 10-item SCS that has been developed by Kalichman et al. [34]. Responses were recorded on a four-point rating scale (1 = not at all like me, 2 = slightly like me, 3 = mainly like me, 4 = very much like me, e.g., “I have to struggle to control my sexual thoughts and behavior”). In this study, the Cronbach’s alpha of this scale was 0.86.

Questionnaire of OSAs. Thirteen items were used to measure participants’ use of the internet for the following purposes: (1) viewing sexual explicit materials (SEM), (2) seeking sexual partners, (3) cybersex, and (4) flirting and sexual relationship maintenance [35]. Viewing SEM was assessed using five items (e.g., visiting erotic/pornographic websites, viewing and downloading erotic/pornographic videos from the internet, reading erotic/pornographic material online), each of which required responses to be rated on a nine-point scale that ranged from 1 (never) to 9 (at least once a day). The other three subscales assessed frequency using a nine-point scale that ranged from 1 (0 times) to 9 (20 or more times). Two items measured the frequency with which the respondents had sought sexual partners as well as the number of sexual partners that they had sought and found online. The frequency of engagement in cybersex was assessed using four items (e.g., masturbating or viewing strangers masturbating in front of a webcam, describing sexual fantasies either through texts or orally). Internet use for the purposes of flirting and sexual relationship maintenance was measured using two items. The Cronbach’s alpha of the entire scale was 0.88 in the study. Higher scores were indicative of more frequent engagement in OSAs.

Additional Questions about IPU. In addition to items that assessed demographic characteristics, a few questions that were related to IPU were also posed to the participants. After providing them with a clear definition of internet pornography, the participants were asked to indicate their age of first exposure to pornography and the duration of time that they typically spent watching internet pornography every week.

##### The Reference Standard—BPS

The BPS, which has been developed by Kraus et al. [26], was used to assess pornography use during the past 6 months. This five-item assessment uses a three-point rating scale (0 = never, 1 = occasionally, 2 = always, e.g., “You find it difficult to resist strong urges to use sexually explicit material.”); a cutoff score of 4 was used to detect problematic pornography use (absolute range = 0–10). Higher scores are indicative of more problematic pornography use. The Cronbach’s alpha of the BPS was 0.84.

#### 2.1.3. Procedure

This online study was conducted through a popular Chinese survey website, namely, Wenjuanxing (www.sojump.com). Adult members of the website received an email with a link that redirected them to the survey website and a brief introduction to our survey. This brief introduction informed the recipients that they were eligible for participation if they had engaged in IPU during the past 6 months (e.g., reading online pornographic content, browsing pornographic websites, sharing/watching pornographic videos or pictures, interacting and flirting with others) and were interested in participating in the survey. A total of 972 valid responses were collected from participants from 110 cities in 28 of the 34 provinces/regions in China (i.e., identified using the internet protocol addresses). As expected, all participants obtained scores that were equal to or greater than 14 on the measure of OSAs (the lowest possible score is 13, and it indicates no prior IPU); this indicated that all of them had engaged in at least one OSA during the past 6 months. Three highly homogeneous samples were required to respond to the three measures of problematic IPU, namely, the PPCS, PPUS, and s-IAT-sex, respectively. Each sample also completed the aforementioned mentioned assessments against which their criterion validity was to be examined. This study was conducted in accordance with the Declaration of Helsinki, and the protocol was approved by the Ethics Committee of the Department of Psychology, Fuzhou University (date of approval, 7 April 2019).

### 2.2. Analysis

Statistical analyses were conducted using SPSS 19.0 (IBM, Armonk, NY, USA) and Mplus version 7 [36]. Item-total correlations were computed to identify items that functioned poorly. CFA was used to test the factor structures of the scales of interest. Maximum likelihood estimation with the Satorra-Bentler correction was used to determine the fit between the data and the factor structures. Model fit was tested by inspecting the following indices: root mean square error of approximation (RMSEA; good: ≤0.06, acceptable: ≤0.08), comparative fit index (CFI; good: ≥0.95, acceptable: ≥0.90), and Tucker-Lewis index (TLI; good: ≥0.95, acceptable: ≥0.90). The reliability of the scales was assessed by computing Cronbach’s alpha coefficients.

To identify possible groups of at-risk pornography users, latent profile analysis (LPA) was used. LPA was conducted using the original dimensions of each scale as explicit variables, and different groups of individuals with problematic IPU were successively divided into two to four categories for model fitting estimation. Sensitivity was defined as the proportion of persons with positive symptoms (as detected by the BPS) and members of the at-risk group (identified through LPA), whereas specificity was defined as the proportion of persons with negative symptoms and the nonproblematic group [37].

### 2.3. Results and Discussion

#### 2.3.1. Validation of the Three Scales

The results of item analysis, CFA, and tests of reliability and convergent validity are shown in Table 2. Item-sum correlations were computed to examine item functioning. The PPCS and PPUS yielded higher coefficients, and both these scales also yielded good fit indices (i.e., CFA) and stronger reliability coefficients. PPCS, PPUS, and s-IAT-sex significantly positively related with SCS, PCQ, OSAs and usage time severally, and PPCS demonstrated stronger convergent validity.

#### 2.3.2. LPA

The results of LPA are shown in Table 3. For PPCS, the Lo-Mendell-Rubin adjusted likelihood ratio test (LMRT) results were significant when the number of classes was 4, and the entropy value was lower. Thus, the classification accuracy was not as high as that of the three-class solution; accordingly, the three-class solution was selected. For PPUS, when the model consisted of three classes, the LMRT results were significant; furthermore, the entropy value was evidently higher than that of the four-class solution. With regard to the s-IAT-sex, the nonsignificant *p*-value that emerged for the LMRT results suggested that the three- and four-class solutions should be rejected in favor of the two-class solution.

With regard to the three groups that emerged for the PPCS and PPUS, the first class obtained the lowest averages across all the scale dimensions; thus, this group was referred to as nonproblematic consumption. The second class obtained moderate scores on all the scale dimensions; therefore, these group members were referred to as low-risk pornography users. The third class obtained the highest scores on all the scale dimensions; thus, this group was referred to as at-risk users. As shown in Table 4, with regard to the two classes that emerged for the s-IAT-sex, class 1 obtained lower scores than class 2 on both the scale dimensions; therefore, they were referred to as the nonproblematic and at-risk groups, respectively (group differences in scores on the specific dimensions are shown in Appendix A).

#### 2.3.3. Sensitivity and Specificity Analysis

The results showed that the sensitivity of the PPCS was 89.66%, which is higher than the values that emerged for the PPUS (i.e., 81.25%) and the s-IAT-sex (i.e., 71.72%). There were differences in the specificity of the three scales, and the values ranged from 85.86% to 94.95%. The PPCS demonstrated greater sensitivity (89.66%), and its specificity was 85.86%. This indicates that approximately 10% of problematic users had been classified as nonproblematic users and that approximately 14% of nonproblematic users had not been identified. In general, the PPCS and PPUS performed better than the s-IAT-sex. Since this study aimed to identify the scale with greater sensitivity in detecting problematic IPU, the PPCS was investigated in greater detail.

## 3. The Qualitative Part: Identification of the Most Accurate Scale

### 3.1. Methods

#### 3.1.1. Sample

We interviewed 22 (20 men; mean age = 27.2) problematic IPU service volunteers (who provide online services on the following website: http://www.ryeboy.org/; average service time = 3.3 years) and 11 therapists (who have worked with individuals with problematic IPU and had more than 3 years of clinical experience).

#### 3.1.2. The Interview Outline

Since the used scales were easy to administer and consisted of close-ended questions, interviews were conducted to examine participants’ perspectives more deeply and comprehensively. The interview guide primarily sought to explore interviewees’ understanding of problematic IPU/addiction and their evaluations of the dimensions of the selected scale. The interviewees were required to rate the importance of the dimensions on a scale that ranged from 1 (not at all important) to 7 (very important).

#### 3.1.3. Procedure

In this study, we primarily explored their understanding of the concept of problematic IPU and the dimensions of the recommended scale. Two psychology graduate students served as the interviewers. At the beginning of the interview, the interviewees were informed about the purpose and significance of the interview and assured of the anonymity and strict confidentiality of their interview data; the interviews were recorded with their permission.

### 3.2. Analysis

The interview recordings were transcribed into verbatim scripts, and participants’ identifying information was concealed. Next, we undertook thematic analysis of the text; in other words, we collated different interviewees’ responses to the same question to create new text. Tree Nodes were established based on the dimensions of the selected scale, and interviewees’ original statements were identified and summarized as a named code. Through this process, NVivo automatically generated statistics for all the references of the texts.

### 3.3. Results

**Figure 1:**
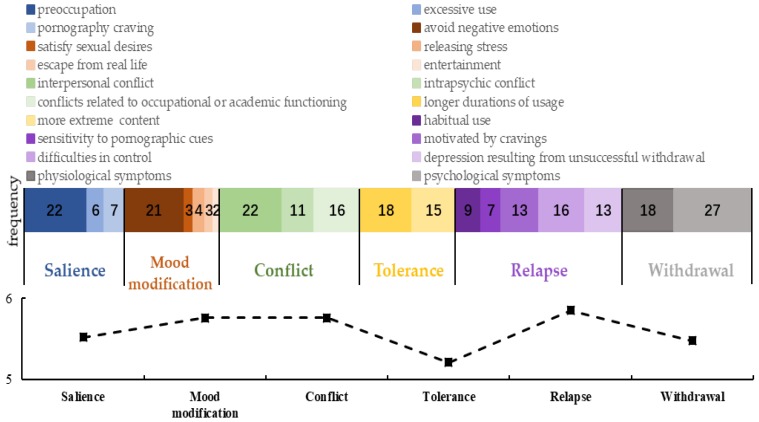
Volunteers and therapists’ frequency of mentioning the dimensions of the Problematic Pornography Consumption Scale, features, and importance ratings for the six dimensions (average scores across 33 interviewees). Note: the numbers in the color blocks represent the frequency of mentions, whereas the polyline represents importance ratings for the six dimensions (range = 1–7).

With regard to the characteristics of problematic IPU, we generated a total of 20 codes by analyzing the interview data. Among these features, preoccupation with IPU (22 mentions), IPU to escape or avoid a negative emotional state (21 mentions), interpersonal conflict (22 mentions), and physiological and psychological symptoms (45 mentions) were most commonly mentioned. Furthermore, the 20 codes were summarized into the six dimensions of the PPCS (see Figure 1).

Instance of the interview:

Interviewer: According to your service experience, what do you think is problematic internet pornography use? In other words, what are the expressions/symptoms of problematic internet pornography use?
Interviewee (service volunteer): They (problematic users) show difficulty controlling the craving for internet pornography (code: pornography carving), they are unable to control their own behavior, for instance, browsing pornographic websites, masturbating while watching porn frequently (code: difficulties in control). Their brains are constantly bombarded with sexual materials (code: preoccupation). If they are not exposed to internet pornography, they will feel uncomfortable, or feel that their heart is empty (code: depression resulting from unsuccessful withdrawal).

After presenting interviewees with the definitions of the six components of problematic IPU and further clarifying their meaning using examples, we presented them with questions “Based on your service experience, do you endorse this structure? Which dimension or dimensions do you think are particularly central to IPU?” Most (>95%) participants endorsed the six dimensions. It also can be inferred from Figure 1 that both volunteers and therapists emphasized the centrality of conflict, relapse and withdrawal in IPU (basing the frequency of mentions); at the same time, they weighted the mood modification, relapse and withdrawal as more important features in the problematic use (basing the important rating).

## 4. General Discussion

Problematic IPU is still a controversial issue; notably, it appears that no real consensus exists regarding the conceptualization and screening tool of problematic IPU. Several scales are available; thus, the assessment of problematic IPU is inconsistent, indicating that findings in this area are not readily comparable. The present study aimed to selected a more sensitive scale to screen problematic IPU, because higher sensitivity implies lower rate of missed diagnosis (i.e., problematic users who have been incorrectly screened as nonproblematic users). Basing on a systematic literature review, three scales were retained. Considering that research with mixed methods combining quantitative and qualitative analyses can enrich and improve our understanding of complicated phenomena [38,39], a quantitative method was used to identify a “more accurate” analysis from the three retained scales. Results of CFA showed that all three scales have good applicability in the wide range of adult groups (age in this case ranged from 18 to 45 years) in three highly homogeneous samples; compared to the other two scales, the PPCS demonstrated greater sensitivity and comparative specificity among samples drawn from the general population (results of the QUAN). Considering that the expression of questionnaire survey is brief and closed, and that the interview can understand the participants’ undefined views more deeply and comprehensively, subsequently, results of QUAL showed that symptoms of problematic IPU proposed by the servers (volunteers and therapists) can be grouped into the six dimensions of PPCS and most of the servers supported the six-factor structure of PPCS.

Among the three scales, the PPCS score was most robustly related to the duration of usage, frequency of engagement in OSAs, and pornography cravings. Problematic IPU can appear under the umbrella of hypersexuality similarly to frequent engaging in various forms of cybersex, intense craving for pornography, and compulsive sexual behaviors [40], insofar that the robust relationship not only demonstrated a higher criterion validity, but also implied that co-screening instruments (i.e., pornography craving, frequency and duration of use, compulsive use) are expected to work as auxiliary screening indicators. Recent studies have revealed that for some people, pornographic use gave rise to their feeling of discord and shame contributing to their conflict of actual sexual materials consumption and their belief; in turn, these feelings of distress and shame may drive a morbid self-perception that they are addicted, but this may not be a real behavioral disorder [41,42]. In order to avoid misjudgment due to the self-perceived problematic use, it is more advisable to combine other supporting scales, and the combination diagnosis indexes of the diversity were selected to screen the prevalence of problematic IPU. In this study, with the higher correlation of PPCS with frequency of OSAs, the PCQ showed that combined with other indicators, it can better screen out problematic use and is more likely to avoid the misjudgment caused by subjective self-perceived addiction.

The more robust psychometric properties and higher recognition accuracy of the PPCS may be attributable to the fact that it has been developed in accordance with Griffiths’s six-component structural theory of addiction (i.e., in contrast to the PPUS and s-IAT-sex). The PPCS has a very strong theoretical framework, and it assesses more components of addiction [11]. In particular, tolerance and withdrawal are the important dimensions of problematic IPU that are not assessed by the PPUS and s-IAT-sex; PPCS is the only instrument that explicitly assesses the “tolerance” component [11,14]. According to the “two-phased” internet pornography addiction model, in which the first step is characterized by an excessive use to internet pornography, and the second functions as a marker by repeated failures to break free from excessive use, despite negative consequences [43]. Items related to information about salience, carving, and tolerance reflect the engagement in internet pornography, corresponding to the first step, whereas items related to withdrawal, relapse, and conflict measure addiction more, corresponding to the second step. Obviously, components of PPCS includes both engagement in pornography and addiction of IPU, which has an intact theoretical framework of addiction.

The PPCS appears to be a more valid instrument for assessing problematic pornography use, has potential application in detecting prevalence concerning problematic IPU or cybersex addiction, and may be useful in assessing treatment outcomes. Our findings indicate that individuals who score high on the PPCS also report frequent engaging in various forms of online sexual activities, intense craving for pornography, and compulsive sexual behaviors. Thus, it appears important for clinicians to be aware of problematic pornography use and its related associations such as pornography craving, compulsive use. Moreover, it is important to note that the scale PPCS is recommended as a screening instruments to identify problematic users in the public and assess the prevalence rather than a diagnostic tool; future studies should further research its validity and cutoff in clinical sample; we also encourage individuals to visit a clinical therapist after being identified with problematic IPU by the use of PPCS.

This study has several limitations. First, data were collected using self-report measures; therefore, the reliability of the results depends on the respondents’ honesty and accuracy of their comprehension of the scale items. Second, the study sample was recruited through an online survey company; therefore, the participants of this study may have been more educated and affluent than the average Chinese person. Furthermore, the study participants primarily lived in the capital/provincial capital, cities, and towns. Third, because the sample consisted of only a small number of non-heterosexual subjects, it was not possible to examine whether the factor structure and meaning of the contents of the PPCS differed across individuals with different sexual orientations.

## 5. Conclusions

The present study showed that the PPUS, PPCS, and s-IAT-sex are promising measures of problematic IPU. However, when sensitivity and specificity were simultaneously examined, the PPCS emerged as a more suitable measure of problematic IPU. The qualitative findings further confirmed that service providers endorsed the underlying structure of the PPCS.

## Figures and Tables

**Table 1:** Group comparisons of the demographic characteristics of the three study samples.

The Scales		PPCS ^1^ (*n* = 317)	PPUS ^2^ (*n* = 332)	s-IAT-Sex ^3^ (*n* = 323)	χ ^2^ (*F*)	*p*
Gender ratio (men/women)		1.39	1.39	1.48	6.92	0.31
Age	Mean ± SD ^4^	24.64 ± 7.39	24.47 ± 7.27	25.31 ± 6.93	1.24	0.29
	Range	18–48	18–45	18–45		
Sexual orientation	Homosexual	1.31%	1.76%	0.91%	2.61	0.11
	Heterosexual	91.52%	91.97%	94.69%		
	Bisexual	7.17%	6.37%	4.40%		
Relationship status	Single	46.78%	42.52%	40.64%	12.85	0.23
	Partnered	14.81%	21.41%	22.90%		
	Engaged	0.90%	1.20%	0.96%		
	Married	37.51%	34.91%	35.60%		
Educational level	Primary school or below	0	0	0	2.99	0.08
	Vocational school	1.24%	0.35%	0.34%		
	Middle school	1.55%	0.67%	0.91%		
	University or college	97.21%	99.08%	98.75%		
Work	Full time	46.10%	45.51%	47.43%	0.39	0.53
	Part time	2.51%	2.68%	4.57%		
	Short-term hired labor	1.64%	1.21%	0.56%		
	Unemployed	49.75%	50.60%	47.54%		
Place of residence	Capital	36.01%	47.37%	43.69%	11.70	0.07
	County town	40.38%	31.02%	35.04%		
	Town	12.01%	10.51%	8.36%		
	Village	11.70%	11.10%	13.01%		
Age of first exposure to pornography		16.21 ± 4.27	16.62 ± 4.69	16.62 ± 4.81	75.86	0.08

^1^ PPCS = Problematic Pornography Consumption Scale, ^2^ PPUS = Problematic Pornography Use Scale, ^3^ s-IAT-sex = Short Internet Addiction Test Adapted to Online Sexual Activities, ^4^ SD = standard deviation.

**Table 2:** Reliability and validity of the three scales.

Scales	Item Analysis	Confirmatory Factor Analysis				α	External and Convergent Validity			
	*r*s (Item-Sum Correlation)	χ ^2^/*df*	CFI ^4^	TLI ^5^	RMSEA ^6^ [90% CI ^7^]		SCS ^8^	PCQ ^9^	OSAs ^10^	UT ^11^
PPCS ^1^	0.62 ***–0.82 ***	210.70/120	0.963	0.952	0.049 [0.038, 0.060]	0.96	0.67 ***	0.70 ***	0.67 ***	0.28 ***
PPUS ^2^	0.66 ***–0.85 ***	90.30/48	0.966	0.953	0.052 [0.035, 0.068]	0.95	0.71 ***	0.66 ***	0.56 ***	0.17 ***
s-IAT-sex ^3^	0.60 ***–0.79 ***	155.55/52	0.923	0.902	0.079 [0.082, 0.109]	0.89	0.73 ***	0.66 ***	0.46 ***	0.22 ***

^1^ PPCS = Problematic Pornography Consumption Scale, ^2^ PPUS = Problematic Pornography Use Scale, ^3^ s-IAT-sex = Short Internet Addiction Test Adapted to Online Sexual Activities, ^4^ CFI = comparative fit index, ^5^ TLI = Tucker-Lewis index, ^6^ RMSEA = root mean square error of approximation, ^7^ CI = confidence interval, ^8^ SCS = Sexual Compulsivity Scale, ^9^ PCQ = Pornography Craving Questionnaire, ^10^ OSAs = online sexual activities, ^11^ UT = usage time. *** *p* < 0.001.

**Table 3:** Fit indices for latent profile analysis of the three scales assessing problematic internet pornography use.

Scales	Classes ^4^	AIC ^5^	BIC ^6^	SSABIC ^7^	Entropy	LMRT ^8^
PPCS ^1^ (*n* = 317)	2	9298.755	9370.174	9309.910	0.959	1154.76 ***
	**3**	**8898.213**	**8995.944**	**8913.478**	**0.940**	404.51 *
	4	8746.574	8870.618	8765.950	0.899	161.63 *
PPUS ^2^ (*n* = 332)	2	5718.021	5767.488	5726.251	0.953	799.82 ***
	3	**5424.503**	**5492.995**	**5435.899**	**0.924**	293.41 **
	4	5348.339	5435.857	5362.900	0.931	83.29
s-IAT-sex ^3^ (*n* = 323)	2	**3652.433**	**3678.877**	**3656.674**	**0.845**	205.41 ***
	3	3588.004	3625.780	3594.062	0.771	66.59
	4	3538.775	3587.884	3546.650	0.824	52.22

^1^ PPCS = Problematic Pornography Consumption Scale, ^2^ PPUS = Problematic Pornography Use Scale, ^3^ s-IAT-sex = Short Internet Addiction Test Adapted to Online Sexual Activities, ^4^ classes = number of latent classes, ^5^ AIC = Akaike information criterion, ^6^ BIC = Bayesian information criterion, ^7^ SSABIC = sample-size-adjusted Bayesian information criterion, ^8^ LMRT = Lo-Mendell-Rubin adjusted likelihood ratio test, *p* = *p*-value associated with the LMRT results. Bold text is the finally selected models. * *p* < 0.05, ** *p* < 0.01, *** *p* < 0.001.

**Table 4:** Comparisons of the accuracy of the three scales.

Scale	Group	Scores on the BPS					Mean	Range
		<4	4 ≤ x < 6	≥6	Sensitivity ^4^	Specificity ^5^		
PPCS ^1^ (*n* = 317)	At-risk (*n* = 29)	3	11	15	89.66%	-	4.60 ± 0.59	1–7
	Low-risk (*n* = 90)	28	34	28	-	-	2.89 ± 0.46	1–7
	Nonproblematic (*n* = 198)	170	23	5	-	85.86%	1.41 ± 0.39	1–7
PPUS ^2^ (*n* = 332)	At-risk (*n* = 48)	9	8	31	81.25%	-	2.43 ± 0.48	0–5
	Low-risk (*n* = 86)	43	25	18	-	-	1.12 ± 0.29	0–5
	Nonproblematic (*n* = 198)	188	8	2	-	94.95%	0.75 ± 0.84	0–5
s-IAT-sex ^3^ (*n* = 323)	At-risk (*n* = 99)	28	26	45	71.72%	-	2.84 ± 0.44	1–5
	Nonproblematic (*n* = 224)	195	19	10	-	87.05%	1.54 ± 0.42	1–5

^1^ PPCS = Problematic Pornography Consumption Scale, ^2^ PPUS = Problematic Pornography Use Scale, ^3^ s-IAT-sex = Short Internet Addiction Test Adapted to Online Sexual Activities, ^4^ Sensitivity = the proportion of persons with positive symptoms and members of the at-risk group that was identified through LPA, ^5^ Specificity = the proportion of persons with negative symptoms and the nonproblematic group.

**Figure A1:**
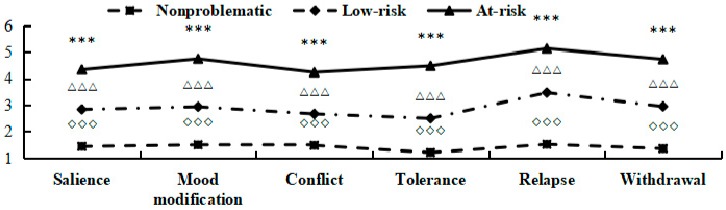
The average scores of the three latent classes based on the dimensions of PPCS. Note: PPCS = Problematic Pornography Consumption Scale, range = 1–7; *** *p* < 0.001 indicate that the score of at-risk group was significantly higher than that of low-risk group; ^△△△^ *p* < 0.001 indicate that the score of low-risk group was significantly higher than that of non-problematic group; ^◇◇◇^ *p* < 0.001 indicate that the score of at-risk group was significantly higher than that of non-problematic group. The same below.

**Figure A2:**
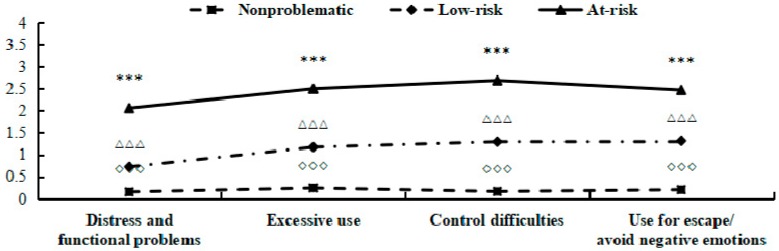
The average scores of the three latent classes based on the dimensions of PPUS. Note: PPUS = Problematic Pornography Use Scale, range = 0–5.

**Figure A3:**
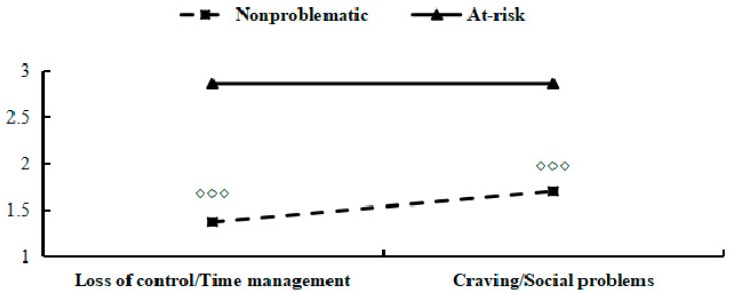
The average scores of the tow latent classes based on the dimensions of s-IAT-sex. Note: s-IAT-sex = short version of the Internet Addiction Test adapted to online sexual activities, range = 1–5.
